# Case report: Antiplatelet therapy on metastatic paraganglioma-associated cutaneous vascular disease and literature review

**DOI:** 10.3389/fmed.2022.1065350

**Published:** 2022-11-17

**Authors:** Yinjie Gao, Yunying Cui, Zhonghui Hu, Yu Wang, Tianyi Li, Yuehua Liu, Anli Tong

**Affiliations:** ^1^NHC Key Laboratory of Endocrinology, Peking Union Medical College Hospital, Beijing, China; ^2^Department of Endocrinology, Peking Union Medical College Hospital, Chinese Academy of Medical Sciences, Peking Union Medical College, Beijing, China; ^3^Department of Dermatology, Peking Union Medical College Hospital, Beijing, China

**Keywords:** pheochromocytoma/paraganglioma, diffuse erythema, tumor-associated cutaneous vascular disorders, antiplatelet therapy, hypercoagulability

## Abstract

**Context:**

Tumor-associated cutaneous vascular disorder induced by PPGL was extremely rare, and the cutaneous manifestations could disappear after removal of the tumors. However, the definite pathological diagnosis and the potential mechanism remained unidentified. We presented a severe cutaneous vascular lesion manifested as diffuse erythema with ulceration and necrosis over the limbs in a female patient with metastatic paraganglioma. Skin biopsy was performed on her for defining the pathological diagnosis and potential mechanism. The patient was diagnosed as vascular disease according to the obvious angioectasia in dermis on cutaneous pathology, which might be caused by PPGL-induced hypercoagulability. We used the antiplatelet therapy with aspirin to treat the PPGL-associated cutaneous vascular disease for the first time, and the cutaneous lesions were relieved and healed gradually, further supporting the diagnosis of vascular disease.

**Conclusion:**

For metastatic PPGL patients like the case we reported, the definite diagnosis by skin biopsy and the early antiplatelet therapy might be effective to the cutaneous lesions caused by the hypercoagulable state of PPGL.

## Introduction

Pheochromocytoma/paraganglioma (PPGL) is a neuroendocrine tumor with various protean manifestations (1). There may be several undetected and unraveled manifestations of this disease. Tumor-associated cutaneous vascular disorder induced by PPGL was first described in the 1970s, and to date, only 16 cases of PPGL-related cutaneous involvement have been reported. Removal of the tumors can lead to complete regression of the cutaneous lesions in these cases (2). However, the definite pathological diagnosis and the evidence for clinical treatment were both limited. Until now, there is no report yet on the effective treatment for severe cutaneous lesions in metastatic PPGL patients who could not receive surgery.

In this report, we presented a metastatic paraganglioma patient with severe diffuse erythema and localized ulcers and curst over the limbs, with suspected association with the hypercoagulability of the tumor. We noted that the cutaneous lesions were relieved and eventually healed gradually, after an antiplatelet therapy with aspirin. We used the antiplatelet therapy with aspirin to treat the PPGL-associated cutaneous vascular disease for the first time, and the potential mechanism behind this and the choice of treatment for PPGL-related cutaneous lesions have been discussed at length in the manuscript.

## Case description

**FIGURE 1:**
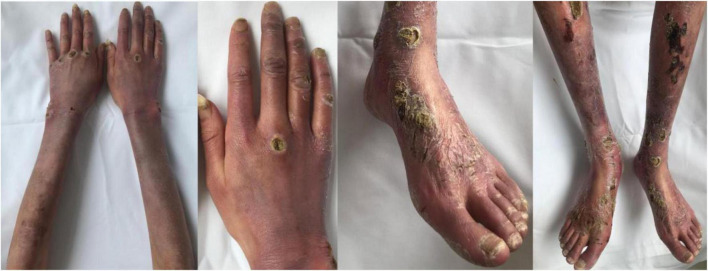
Diffuse erythema with severe ulceration and necrosis on the patient’s arms, hands, legs, and feet.

In October 2016, a 19-year-old Chinese girl was admitted to a local hospital with an elevated blood pressure (180/140 mmHg), accompanied by paroxysmal headache and sweating. Her ^18^F-fluorodeoxyglucose positron emission tomography/computed tomography (^18^F-FDG-PET/CT) scan revealed an abdominal tumor of size 5.3 × 4.8 cm and SUV_*max*_ 8.5, along with multiple liver metastases. The abdominal mass was diagnosed as paraganglioma on biopsy pathology. She had not received any other therapy than long-term phenethylamine for controlling the symptoms. From December 2019 to October 2020, she underwent chemotherapy with cisplatin for 10 cycles intermittently, but there was little change in the abdominal tumor or liver metastases. In April 2021, cutaneous lesions manifested as diffuse erythema with ulcers, obvious pain, and tenderness appeared over the limbs (Figure 1), but without swelling, fever, or joint pain. Upon treatment with methylprednisolone at the dose of 16 mg qd for 1 month and subsequently, with 20 mg qd for another 1 month, the cutaneous lesions showed no improvement, and hence glucocorticoid was discontinued.

**FIGURE 2:**
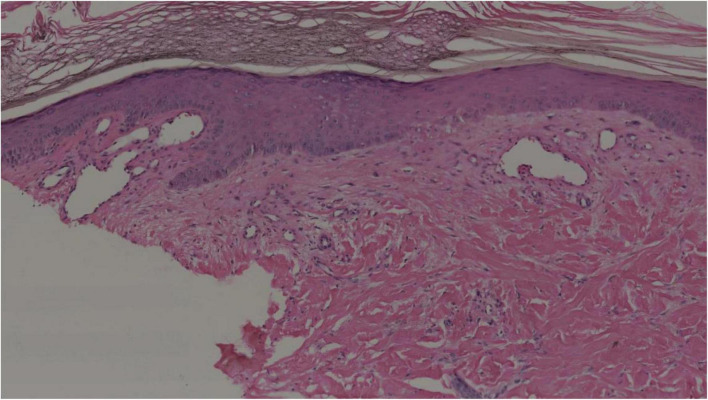
Histopathological findings of the cutaneous lesions on the left leg. Hyperkeratosis, acanthosis, and obvious angioectasia in the dermal papilla and mild lymphocytic infiltration around the dermal vessels (hematoxylin–eosin, original magnification 10×).

In September 2021, the patient was referred to our hospital for further examination. Her physical examination indicated a blood pressure of 140–150/90–100 mmHg. Her body mass index (BMI) was 16.7 kg/m^2^. The cutaneous lesions manifested as diffuse erythema with severe ulceration and necrosis on her limbs. Her laboratory tests indicated a WBC count of 10.5*10^9^/L [(3.5–9.5)*10^9^/L], HGB of 106 g/L [(110–150) g/L], a high platelet (PLT) count of 673*10^9^/L [(100–350)*10^9^/L], abnormal coagulation function with a high D-Dimer level of 596 ng/ml [(0–253) ng/ml]. Endocrine-related hormone tests indicated significantly increased catecholamine levels [NMN: 399.38 nmol/L (<0.9 nmol/L); MN: 0.61 nmol/L (<0.5 nmol/L); 24-h urinary NE: 9567.1 μg/24 h (<76.9 μg/24 h); 24-h urinary E: 2.3 μg/24 h (<11 μg/24 h), 24-h urinary DA: 5354.1 μg/24 h (<459.9 μg/24 h)]. A skin biopsy taken from the lesions on her left leg revealed significant hyperkeratosis and acanthosis. There was obvious angioectasia in the dermal papilla and mild lymphocytic infiltrate around the dermal vessels (Figure 2). The characteristics of cutaneous lesions were consistent with that of cutaneous vascular disease, and the patient accordingly received anticoagulant therapy.

**FIGURE 3:**
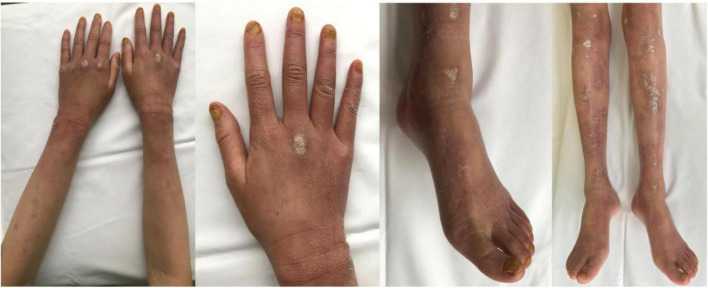
Evident regression of the cutaneous lesions of the patient’s arms, hands, legs, and feet after the antiplatelet therapy.

The patient took rivaroxaban, but epistaxis and ulcer bleeding occurred soon after. Therefore, rivaroxaban was withdrawn and switched to the antiplatelet drug aspirin at the dose of 25 mg qd. After the treatment with aspirin for 2 months, her cutaneous ulcer healed and the diffuse erythema disappeared, with only atrophie blanche and skin hyperpigmentation left (Figure 3).

Genetic screening of the genomic DNA from peripheral blood leukocytes of the patient was conducted through targeted next-generation sequencing (NGS) involving 20 genes (*NF1, VHL, RET, SDHA, SDHB, SDHC, SDHD, SDHAF2, MAX, TMEM127, FH, KIF1B, BAP1, IDH1, EPAS1, EGLN2, EGLN1, EGLN3, HRAS*, and *MDH2*). A pathogenic heterozygous variation of *SDHB* (NM_003000.3), c.C136T (p.Arg46Ter) was detected by using targeted NGS. The patient was prescribed chemotherapy with temozolomide (TMZ) after the cutaneous lesions cured. Before the treatment was performed, catecholamines and metabolites were tested to reveal the following results: NMN: 891.39 nmol/L; MN: 0.52 nmol/L; 24-h urinary NE: 27255.7 μg/24 h; 24-h urinary E: 3.6 μg/24 h, 24-h urinary DA: 6907.8 μg/24 h. The patient was found to demonstrate multifocal abdomen, liver, and bone lesions on contrast-enhanced CT and ^68^Ga-DOTATATE-PET/CT. She was then put on TMZ at 150 mg/m^2^/day on days 1–5 in a 28-day cycle. In the second and subsequent cycles, she took TMZ at 200 mg/m^2^/day (on a 5/28-day regimen). After 6 cycles, the levels of catecholamines and metabolites decreased significantly (NMN: 286.35 nmol/L; MN: 0.21 nmol/L; 24-h urinary NE: 7487.5 μg/24 h; 24-h urinary E: 3.8 μg/24 h, 24-h urinary DA: 323.7 μg/24 h), and CT suggested that the lesions have diminished in some degree. The patient tolerated TMZ well, and only suffered from mild nausea during the medication. No bone marrow suppression and abnormal liver and kidney functions were recorded.

## Discussion

Researches showed that paraneoplastic vascular disorders could be found on PPGL patients. We reviewed the cases of PPGL demonstrating cutaneous involvement in the previous studies (Table 1). In half a century, reports on PPGL-associated cutaneous disorders have been scanty across the globe, and the cutaneous lesions manifesting as purpura, erythema, or even partial necrosis tend to involve limbs, hands, and feet. Some studies reported that the outcomes of patients after removal of the PPGL tumors were remarkably favorable with a complete regression of the cutaneous lesions (3–7).

**TABLE 1:** Summary of the cases of pheochromocytoma/paraganglioma with cutaneous involvement in the literatures.

References	Age	Gender	Diagnosis of PPGL	Cutaneous presentation	Skin biopsy	Treatment	Prognosis
Morales et al. (34)	NA	NA	PCC	Skin rash	NA	NA	NA
Sjoerdsma et al. (14)	63	Male	Metastatic PCC	Striking peripheral cyanosis of the hands, feet, and prepatellar regions, and ulcerated and sloughing skin of some of these areas	NA	α-MPT	Regression of the symptoms
Ledingham et al. (3)	32	Female	PCC	Blotches on the feet	Necrotizing vasculitis	Tumor resection	Regression of the symptoms
Sheps et al. (7)	41	Female	PCC	Livedo reticularis of the lower limbs and infarctive and purpuric skin lesions over the feet and ankles	Skin infarction	Tumor resection	Regression of the symptoms
Dallocchio et al. (8)	33	Female	PCC	Cutaneous abnormalities with erythemato-macular eruption on dorsalis faces of both hands	Non-specific capillaritis and oedema	NA	Regression of the symptoms
Sato et al. (4)	32	Male	PCC	Erythema nodosum-like eruptions of the lower limbs	NA	Tumor resection	Symptoms persisted
Callen et al. (13)	32	Male	Bilateral PCC	Purpuric lesions over the knees, thighs, and penis	NA	Glucocorticoids; tumor resection	Symptoms persisted; regression of the symptoms
Schiraldi et al. (5)	NA	NA	PCC	Purpuric lesions over legs, and arthralgias	NA	Tumor resection	Cutaneous lesions disappeared; cryoglobulins persisted
Guilhou et al. (35)	NA	NA	PCC	Partial necrosis of the fourth toes	NA	NA	NA
Khan et al. (36)	46	Female	PCC	Without detailed description, Behçet’s disease related symptoms	NA	NA	NA
Núñez et al. (37)	47	Female	PCC	Successive rashes of erythematous and pruriginous cutaneous lesions of the lower limbs	Erythema nodosum	NA	NA
Mark et al. (15)	24	Male	PCC	A fixed livedoid rash on back and limbs	NA	Tumor resection	Regression of the symptoms
Weiller et al. (6)	41	Male	PCC	Purpura on the wrist, ankle and lower limbs	Vasculitis	Tumor resection	Regression of the symptoms
Kalhan et al. (16)	54	Female	PCC	A diffuse mottled vasculitic rash (livedo reticularis) on lower half of the body and limbs	NA	Waiting for surgery	Died of PCC crisis
Zajac et al. (17)	49	Transgender female	Metastatic PGL	Livedo reticularis, which later progressed to areas of necrosis	NA	Palliative care	Died of significant deconditioning and cachexia
Bouslama et al. (2)	30	Female	PCC	Reticularis livedo in the four limbs, ulcers in both knees and in the 3rd metacarpo-phalangeal articulations and necrotic lesions in both calcaneal tendons and in the right toes	Non-specific chronic dermatitis	Tumor resection	Regression of the symptoms

The diagnosis of cutaneous lesions on some patients was PPGL-related cutaneous vasculitis, which suggested to be induced by catecholamine excess (6, 8). In addition to the skin involvement, catecholamine has been reported to be associated with the pathogenesis of large vessel vasculitis, such as aortoarteritis and the central nervous system (CNS) vasculitis (9–12). The potential mechanism behind this may be that the catecholamine excess status results in direct damage to the vessel wall as well as triggering of autoimmune mechanism, both of which act in conjunction to produce vasculitis in large and small blood vessels (11, 13). However, the cutaneous pathology of most cases was absent, and only two patients we reviewed presented with vasculitis by the skin biopsy.

Except for the surgical removal of the tumor, drugs that inhibit catecholamine secretion or treat vasculitis may have a certain effect on the cutaneous lesions. Engelman et al. have presented a metastatic PPGL patient with cutaneous lesions responded well to alpha-methyltyrosine (α-MPT), an inhibitor of catecholamine biosynthesis (14). In the previous studies, cases with catecholamine-induced vasculitis have been treated with glucocorticoids or/and cyclophosphamide and achieved improvement in the symptoms, although their final resolution was obtained from the excision of the PPGL tumors (9, 12).

However, there were still some cases with cutaneous lesions that were difficult to explain by catecholamine-induced vasculitis. Researchers reported several PPGL cases suffered from the livedo reticularis (2, 7, 15–17), and the potential mechanism was considered as the hypercoagulability and platelet aggregation which induced by the tumors of PPGL (18, 19). However, the evidence to support the diagnosis was limited. Except for two patients with pathological description as non-specific chronic dermatitis and skin infarction, respectively, there was no definite pathological diagnosis of other patients’ cutaneous lesions. Meanwhile, no experience of drug treatment in how to treat the PPGL-related cutaneous lesions caused by hypercoagulability has been found.

In the previous studies, arteriovenous thrombosis, pulmonary embolism, and ventricular thrombus have also occurred in PPGL patients, wherein PPGL-related hypercoagulability was considered as the causative mechanism (20–26). And after anticoagulant dabigatran or heparin therapy, the thrombus could disappear, showing that anticoagulant drugs might be effective on the hypercoagulability of PPGL (27–33).

The cutaneous biopsy pathology of the present patient was definite and it showed that there was obvious angioectasia in the dermal without leukocytoclasis or true vasculitis, which was considered as the vascular disease caused by hypercoagulability and platelet aggregation. On the other hand, patients with vasculitis may response to the treatment of glucocorticoids or immunosuppressants, but it is ineffective for the present patient. Her cutaneous lesions were eventually relieved and even healed gradually with the aspirin antiplatelet therapy, which was consistent with the therapeutic response of cutaneous vascular disease. We used the antiplatelet therapy with aspirin to treat the PPGL-associated cutaneous vascular disease for the first time, and for metastatic PPGL patients like the case we reported, the early antiplatelet therapy might be effective to the cutaneous lesions caused by the hypercoagulable state of PPGL.

In summary, we presented a metastatic paraganglioma patient with severe cutaneous vascular disease identified by skin pathology, who demonstrated gradual regression of the symptoms after the antiplatelet therapy. PPGL-related cutaneous vascular involvement is extremely rare, which may be resolved after tumor resection. For patients who could not receive the surgery, antiplatelet therapy is effective on cutaneous lesions induced by the hypercoagulable state of PPGL.

## Data availability statement

The original contributions presented in this study are included in the article/supplementary material, further inquiries can be directed to the corresponding authors.

## Ethics statement

Written informed consent was obtained from the individual(s) for the publication of any potentially identifiable images or data included in this article.

## Author contributions

AT and YL designed the study. YG, YC, and ZH collected the clinical data. YC, YW, and TL participated in the diagnosis and treatment of the patient. YG and AT wrote the manuscript. All authors read and approved the final manuscript.
